# Revealing the zone of possible agreement between parties in conflict: An application to Israeli-Palestinian peace agreements

**DOI:** 10.1093/pnasnexus/pgae581

**Published:** 2025-01-21

**Authors:** Elisa Cavatorta, Ben Groom, Gilead Sher

**Affiliations:** Department of Political Economy, King’s College London, London, WC2B 4BG, United Kingdom; Department of Economics, University of Exeter, Exeter, EX4 4PU, United Kingdom; Baker Institute for Public Policy, Rice University, Houston, TX 77005, USA

**Keywords:** peace agreements, zone of possible agreement, Israelis and Palestinians

## Abstract

After Hamas’ attack on 2023 October 7 and Israel’s subsequent war, a pressing question is the nature of a postwar peace agreement. Peace negotiations often become deadlocked due to difficulties in identifying mutually advantageous agreements. A large-scale survey task and method is developed to identify the strength of preference for components of potential peace deals and changes to the status quo. Analyzing pre-October 7 representative samples of Israelis and Palestinians reveals a Zone of Possible Agreement, demonstrating shared preferences for deals that improve daily life. Violence exposure hampers compromise among Israelis, emphasizing the importance of abstaining from violence for conflict resolution.

**Significance Statement**

In multi-issue peace negotiations, finding mutually acceptable agreements is complex, and understanding public acceptability of hypothetical deals is essential. Traditional public opinion surveys fall short in identifying viable compromises. This paper presents an approach to assess preferences for potential deal components and the set of agreements both parties prefer over the status quo: the Zone of Possible Agreements (ZOPA). Using 2022 data from representative Israeli and Palestinian samples, we evidenced a ZOPA: of 256 potential agreements, 55 are rated superior to the status quo by both groups. The most favored deals include practical changes that could improve daily life on both sides. Additionally, exposure to violence hampers compromise prospects among Israelis, underscoring that progress hinges on the cessation of hostilities.

## Introduction

After the trauma inflicted on Israel by Hamas’ massacre on 2023 October 7 and the devastation in Gaza resulting from Israel responding with war on Hamas and Islamic Jihad, a key question in the mind of many concerns the “day after” the war ends: what sort of peace agreement, if any, would Israelis and Palestinians find mutually acceptable? Short of the dream that the diplomatic process that failed for over three decades will suddenly succeed, serious re-thinking about peace agreements that resolve the contentious issues is required and needed more now than ever before.

Designing peace agreements is a complex process, all the more so in intractable conflicts with numerous disputed issues. When parties do negotiate, peace negotiations frequently become deadlocked because the parties are not able to identify mutually advantageous agreements. Even when such configurations exist, at least in principle, they are often not immediately visible. Finding mutually acceptable agreements requires understanding of the ordering of priorities of one’s own group, the acceptable give-and-take one party is willing to engage in to attain a deal, the priorities of the other party and tradeoffs they are likely to agree to.

Understanding the acceptability of peace agreements to the public is important for the peace negotiation process. Public opinion matters because it informs political leaders’ decisions about the timing of negotiations, their mode (e.g. whether they are held in secret or not, (1)) and the concessions. Leaders who act against strong public opinion risk losing political support. These considerations repeatedly appear in the history of the Middle East peace talks. A well-known example illustrating these considerations in leaders from both sides comes from the Clinton-led peace talks in late 1999. Ehud Barak, Israel’s Prime Minister at the time, had a change of heart regarding the agreement with Syria despite his reported willingness to concede on Israel’s withdrawal from the Golan Heights. It is reported he said: “I can’t do it. My people won’t understand. It’s all too quick. I have to prepare my public for a full withdrawal from the Golan and I have to take time” ((2), p. 78). Similarly, Arafat was reported saying in Camp David: “A billion Muslims will never forgive me if I don’t receive full sovereignty in East Jerusalem. I do not have a mandate to compromise. It’s not me, it’s the entire Muslim world,” ((3), p. 84). Both examples underscore the importance of public opinion in shaping leaders’ negotiating positions. Knowledge of public opinion on both sides helps negotiators address the core concerns and grievances of the population. This can lead to more effective conflict resolution tactics and trust building techniques (4, 5). By addressing the legitimate concerns of the majority, the agreements can undermine the narratives of those who seek to derail the peace efforts (6, 7).

Public opinion also matters for the outcomes of negotiations and the prospect of success of peace agreements over time. Research shows that negotiations that are more inclusive and take due understanding of public opinions makes peace agreements more effective and sustainable (e.g. (8–10)). Public referendums in both Northern Ireland and the Republic of Ireland were crucial in legitimizing the agreement and ensuring broad support across communities (11, 12). Moreover, agreements that are supported by the public are more likely to be implemented effectively (13).

Yet, public consultation on the design of prospective peace agreements is fraught with difficulty and traditional ways of gathering public preferences are often inadequate in this context. Public opinion surveys on support for the peace process and acceptability of negotiations play an important role in summarizing what people think and want. Yet, traditional public opinion surveys are ill suited to inform about the acceptability of peace deals for several reasons. First, questionnaires that ask whether one supports peace negotiations cannot speak to what compromises are acceptable or unacceptable. Second, even when respondents express acceptance or rejection of a particular peace deal configuration, such as the “two-state” solution, it does not necessarily imply that no other configuration is acceptable. Questions on support for specific peace deal configurations need to be carefully worded because details matter and respondents may have different ideas about how details left implicit are resolved. For example, supporting a “two-state solution” does not explicitly outline the type of freedom of movement implied for labor and goods. Thirdly, there could be numerous compensatory combinations between components of peace agreements which result in as many peace deal configurations, making direct survey questions impractical. Lastly, traditional surveys typically struggle to disentangle people’s valuations of the content of an agreement from people’s reactions to the way the negotiation process develops.

In this paper, we design a task suitable for surveys that addresses these shortcomings. The task identifies the components of potential peace deals regarded as most important for each side, the relative strength of preferences for them and the strength of support for agreements that deviate from the status quo. The task overcomes the difficulty of traditional questionnaires. We implement it in two nationally representative samples of Palestinians living in the West Bank and Gaza Strip and Israelis living in Israel and the occupied territories. We exploit the bilateral nature of our analysis to visualize the Zone of Possible Agreement (ZOPA): the set of agreements preferred by both groups to the status quo; and the Pareto frontier of peace deals: the set that maximizes the gains achievable by combining concessions and demands on components of a peace deal. We also visualize the zones where unacceptable agreements lie.

We then study how the experience of violence among respondents alters support for prospective peace agreements. This information is important to inform campaigns that tries to support peacemaking efforts, and are crucial after the heights of violence on and after October 7th. Previous studies suggest that violence exposure can harden public opinions about the perceived enemy (14), reduce support for peace, at least in the short term (15), and makes retaliatory inclinations more likely (16). However, previous studies lack evidence on why violence exposure makes support for peace more difficult. Are violence-exposed people rejecting compromise altogether? Or do they become more sensitive to certain concessions? The method described here is able to answer these questions.

## Method: finding the mutually acceptable agreements

In this method, individuals are asked to rank hypothetical peace agreements based on their preference. These peace agreements comprise of “components” representing different aspects of the conflict. Each component signifies either maintaining the current situation (the status quo) or introducing a change from the status quo. Consequently, configurations of peace deals are a mix of these binary “components,” representing variations from or continuations of the existing status quo. We manipulate these combinations experimentally to ensure that each respondent receives a set of peace deals with orthogonal components. This approach enables the causal assessment of the strength of preference for various components within hypothetical peace agreements and their relative desirability. Preferences for individual components are estimated for Israelis and Palestinians, and these preferences are then aggregated for each potential peace agreement. This aggregation identifies peace agreements that are preferred over the status quo, those mutually acceptable to both parties: the ZOPA, and among them, the “best” agreements that achieve the highest gains for both parties, as well as “fairer” agreements, that distribute gains equally. Agreements acceptable only to one party and those unacceptable to both are also identified.

In this application, each peace deal comprises of eight components. The choice of a total number of eight components was driven by methodological considerations of statistical ability to estimate the strength of preference for each component causally (i.e. not confounded), power calculations, and feasibility tests, with the understanding that comparing and ranking multiple deals with eight components was feasible for respondents based on field tests (details reported in Supplementary Material, Sections A and B). These eight dimensions of the conflict were selected based on their significance according to public opinion surveys in the region (e.g. https://www.pcpsr.org/, The Peace Index, The Israeli Voice Index, https://en.idi.org.il) and interviews with scholars from the region (further details on issue selection are in the Supplementary Material, Section C). The components include important topics such as Jewish settlements, the recognition of Israel as a nation-state for the Jewish people, the existence of an independent Palestinian State, freedom of movement, right to access the holy sites, the location of capital cities, treatment of prisoners, allocation of water rights. Table 1 outlines the specific wording of each component, which can be either a variation from the status quo (left column) or a continuation of the status quo (right column), each of them supplemented with an explanation Supplementary Material, S1. From a peace deal’s implementation point of view, all components can occur independently: no component is a prerequisite to another, a consequence of or precludes the occurrence of another component.

**Table 1.** Components of peace agreements: Respondents had access to a more detailed explanation of the components and their levels in the survey itself.

Component	Change from status quo	Status quo
1	Freezing of all settlement building, evacuation of those inside the West Bank. Settlements adjacent to the 1967 line become part of Israel.	Israel’s settlement building continues
2	Palestinians recognize Israel as the nation-state of the Jewish people	Palestinians do not recognize Israel as the nation-state of the Jewish People
3	An independent Palestinian State over the West Bank, Gaza and East Jerusalem with equitable (1:1 in value) land swaps with Israel and no Israeli military presence	The civil and military jurisdiction over Israel, the West Bank and Gaza remains as today
4	Freedom of movement for people (no checkpoints/permits), vehicles and goods between West Bank, Gaza and State of Israel for both Palestinians and Israelis	Current freedom of trade between West Bank, Gaza and State of Israel. Permit system for labor and vehicles
5	Unrestricted right to access to holy sites and freedom of worship for anyone	Current restricted rights to access to holy sites and pray
6	Palestinian capital in Jerusalem’s Arab-majority neighborhoods and Israeli capital in Jewish-majority neighborhoods. Old City is undivided	Israeli capital in West and East Jerusalem and Palestinian capital de facto in Ramallah
7	Mutual amnesty and release for an agreed number of current prisoners in Israeli and Palestinian jails	Current practices of imprisonment, pretrial detention and occasional prisoner release, continue
8	Water rights in proportion to the population: 60% Israel, 40% Palestinian Authority	Oslo II water rights (the same as today): 71% Israel, 29% Palestinian Authority

These details and the rationale for the selection of components can be found in Supplementary Material, Section C and Fig. S1.

All components, whether expressed as a change from the status quo or a continuation, are purposefully described in objective and concrete terms (with explicit descriptions, see Fig. S1) to avoid the pitfall that support on the broad “issue” masks disagreement on how the issue is resolved in practice. Moreover, we carefully avoided nomenclatures and expressions that, despite being in common usage, can be interpreted differently by different people (such as “two-state solution,” “multinational arrangements,” and “economic peace”).

With eight issues in each peace deal, there are $2^{8}=256$ possible deals. Given the impracticality of asking respondents to rank all 256 possible deals, we employed an orthogonal fractional (block) design (17). This design optimally reduces the 256 possible deals to 8 blocks of 8 peace deals each, allowing respondents to rank a manageable subset of peace agreements while still enabling the reliable estimation of the average causal effects of each component.

In practice, the respondent task proceeds as follows: each respondent is randomly allocated to a block. Each block contains eight hypothetical deals. The respondent is then shown four deals, randomly selected from the eight, and visualized as physical or virtual cards (see Supplementary Material, Section E and Figures therein) with each component explained by a tool-tip or the enumerator: the respondent is asked to compare and rank the deals on a “preference rack” from the most preferred to the least preferred. Then, the remaining deals are shown to them one by one in a random order. The respondent is asked to add them to their ranking. The ranking can be modified by moving deals along the rack until the final ordering is confirmed by the respondent. There is no time limit. The sequential way in which deals are shown makes the task easier. When the ranking of the eight deals is confirmed, the respondent is shown a nineth card, representing the status quo, and asked to add it to their ranking according to their preference.^a^

The ranking exercise combined with fractional design has a number of features that represent advances on previous conjoint experimental designs and make it particularly suitable for multiattribute and multiparty applications like ours.

First, the ranking approach provides more information on the structure of preferences compared with “pairwise-choice” designs—which ask respondents to choose (or vote for) one option among two (e.g. (18–20)—and “rating” designs—which ask respondents to rate one choice against another on a grading scale (e.g. (21)). Ranking of all deals in a set, as in this study, provides information on the *relative* preferences over all alternatives. For example, because the ranking approach ensures that all comparisons are made within the same set, the decision of a respondent reveals not only the first best among the options, but also the second best, the third best and so on. These preferences are not necessarily visible in pairwise choices where the “most preferred” option is chosen, unless all pairwise comparisons are made.^b^

Second, ranking of all deals in a set explicitly reveals which deal is “best” or “worst” (most preferred or least preferred) for each individual, without requiring modeling assumptions, e.g. on the shape of the utility function, and it allows the study of the positioning of specific deals of interest within the ranking. This is not possible in designs using pairwise comparisons of a random set of deals, in which each respondent sees different sets. Third, ranking, as opposed to rating, requires less stringent assumptions about the comparability of preferences across individuals. Ranking only assumes comparability of order of preferences (ordinality) rather than comparability of the cardinal values associated with a rating scale.^c^ Fourth, and unlike previous studies, including the ranking of an explicitly defined status quo for all respondents avoid imposing the assumption that everyone has a preference for an agreement.^d^ The rank position of the status quo can be interpreted as a stated-preference measure of the desirability of change from the status quo for each individual. To identify acceptable deals the only requirement is that they are preferred over the status quo by each party. Since both parties observe and rank the same peace deals and the same status quo, this also makes possible to compute measures of support for any specific deal in comparison to the status quo. Fifth, by design, each respondent is presented with a set of deals with uncorrelated components. This allows to study variations of preferences in subgroups causally since subgroup analysis does not compromise the orthogonality of the design.^e^

We assume that the individual rankings of deals reflect ordinal rankings of preference and the desirability of a peace agreement can be represented by an utility function $u_{nj}$, for individual *n* and peace deal *j*, which depends on a vector of agreement components $x_{j}^{\prime }$ and their desirability, and an error term.^f^ Under the assumption that errors follow an extreme value type I distribution, the probability of choosing an alternative is proportional to its utility relative to the sum of utilities of all available alternatives, and it can be written in the multinomial logit form (22–24). The joint probability of a ranking (i.e. from the top position r=1 to the last r=R) can be written as a product of the logit probabilities and estimated by maximum likelihood.^g^


$$\begin{array}{rl} & \text{Pr}\left[u_{r=1}>u_{r=2}>u_{r=3}>,\ldots ..,>u_{r=R}\right]\\ & \;=\text{Pr}[u_{r=1}>\max(u_{r=2},\ldots ,u_{r=R})]\\ & \;\times \text{Pr}[u_{r=2}>\max(u_{r=3},\ldots ,u_{r=R})]\cdots \text{Pr}[u_{r=R-1}>u_{r=R}]\\ & \;=\prod_{\;j=1}^{R}\left[\frac{\exp\;(V_{j}(x))}{\sum_{m=h}^{R}\exp\;(V_{m}(x))}\right]. \end{array}$$

We assume that preferences for peace deals are linear and additively separable in components. We assume that respondents are able to make tradeoffs between components. Additive separability is a plausible assumption since all components represent attributes of potential peace deals that can be implemented separately and independently (see Table 1). The parameters of interest are the vector *β* in $V_{j}(x)=x_{j}^{\prime }\beta$. Each component has an associated parameter which can be interpreted as the expected *difference* in utility for Israelis or Palestinians when a deal’s component is changed from the status quo to an alternative arrangement. The size of the coefficients identifies the relative strength of preferences for the change, with utility as the common metric (the Supplementary Material, Section F discusses methodological considerations regarding the comparability of preferences between components and between societies). Different types of people may have special preferences for specific combinations of components. We analyze this heterogeneity in interdependent preferences in a separate work. The unconfounded main (i.e. average) effects at the sample level are however of primary interest. The parameters of the main effects can be aggregated to yield the desirability of each deal *compared* with the status quo, for both parties in the conflict. This provides the “coordinates” to map each agreement on the utility space, with the utility of the status quo normalized at zero. Peace deals mutually acceptable to both parties are those that yield higher utility compared with the status quo (i.e. are preferred to the status quo) for both parties. Unacceptable deals are those that yield negative utility to one or both parties.

## Data

We collected data from representative samples of Israelis and Palestinians, during approximately the same period of time (end of March 2022 to May 2022), and using the same design. The study received ethics approval from the IRB of the London School of Economics (reference: 07832). Informed consent was obtained from all participants. Due to low levels of computer literacy among the Palestinian population, we adopted an in-person field interview with Palestinians carried out by a professional survey organization^h^ on a sample representative of the Palestinian population in terms of geographical district of residence, gender and age distribution (n=1,197). Israeli respondents were drawn from the database of an Israeli poll company^i^ and interviewed through an interactive online web application that we created.^j^ We set quota on participation and used a greedy algorithm of (25) to generate a sample of 679 Israelis that matches as closely as possible the census statistics on ethnicity (Arab and Jews), district of residence, gender and age distribution from the Israel’s Central Bureau of Statistics. Table S3 shows the descriptive statistics of the samples along with the reference Census benchmark statistics.

For both samples, we used similar instructions and visual devices to make comparisons and ranking of peace deals intuitive to respondents and appropriately designed for each implementation mode. We designed physical cards for the on-the-field application and comparable virtual cards for the online application (see Supplementary Material, Section E). What makes this design compelling is the collection of arguably complex information using visual instruments that make a quantitative task intuitive and easy to complete for many. This is confirmed by the small percentages of people who provide invalid responses. We embedded two quality requirements: (i) Card sequencing and (ii) Task’s completion time. (i) We numbered the cards to check whether individuals ranked them in numerical order (e.g. from card 1 to 9 or vice versa) or in the exact random order in which the cards were presented. In the Palestinian fieldwork-likely the more complex of the two due to lower literacy levels-only three respondents ranked the cards in a numerical sequence. In contrast, 12 respondents in the Israeli sample exhibited this pattern. (ii) We considered responses valid if the task was completed in at least 240 s. This threshold was informed by pilot testing of the interactive web application, where it took 240 s to read the instructions and arrange a larger set of 16 numbered cards in an increasing (or decreasing) order (based on the card number rather than preferences). Responses that failed to meet either of these criteria (i) or (ii) were excluded from the analysis. The median task’s completion time is 7 min to rank eight cards.

## Acceptability of deals

**Fig. 1.**
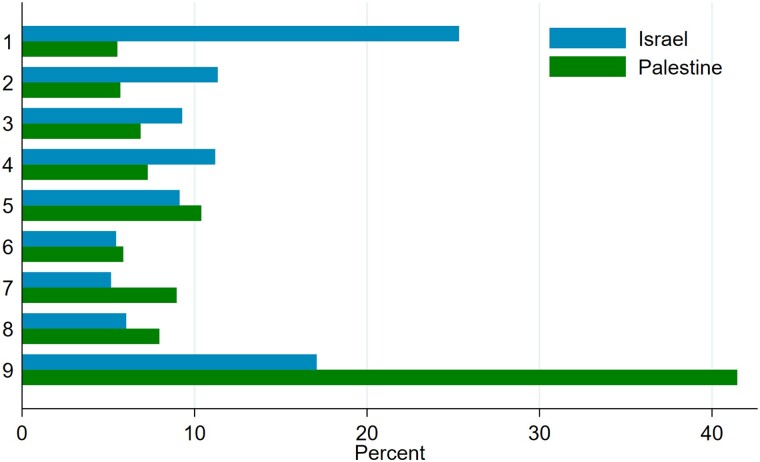
Ranking position of the status quo scenario: 1 (first) = most preferred to 9 (nineth) = least preferred. The status quo is the same scenario for all respondents and all respondents ranked the status quo.

All respondents ranked the status quo in addition to the eight peace deals. Therefore, the position of the status quo in the ranking of deals can serve as a general, unconditional measure of perceived deals’ acceptability. In Fig. 1, it is evident that 75% of Israelis and 95% of Palestinians find at least one deal preferable to the status quo. There is a noticeable difference in the mode of the distribution of the status quo position in the ranking between the two samples. For Palestinians, 41% rank the status quo as the least preferred scenario, making it the most frequently chosen position. In contrast, the Israeli sample appears polarized, with 25% ranking the status quo as the most preferred scenario and 17% ranking it as the least preferred. The demographic composition of these groups differs significantly. The 25% of Israelis favoring the status quo are predominantly male (60% compared with the expected 50%), Jewish Israelis (86% compared with the expected 81%), relatively young (median age 37.5 vs. expected 43 year old in the sample). On the other hand, the 17% who rank the status quo last are older (median age 44), predominantly female (64%), and include a higher proportion of Arab Israelis (56% instead of expected 19%).

In the Palestinian sample, the demographic composition of those who rank the status quo as the most preferred compared with those who rank it as the least preferred scenario is similar in terms of gender composition (gender ratio are equal), mean age (39 years old in both cases: the sample average), and geographical origin of the respondents (as expected in the sample).

## Visualizing the zone of possible agreements

**Fig. 2.**
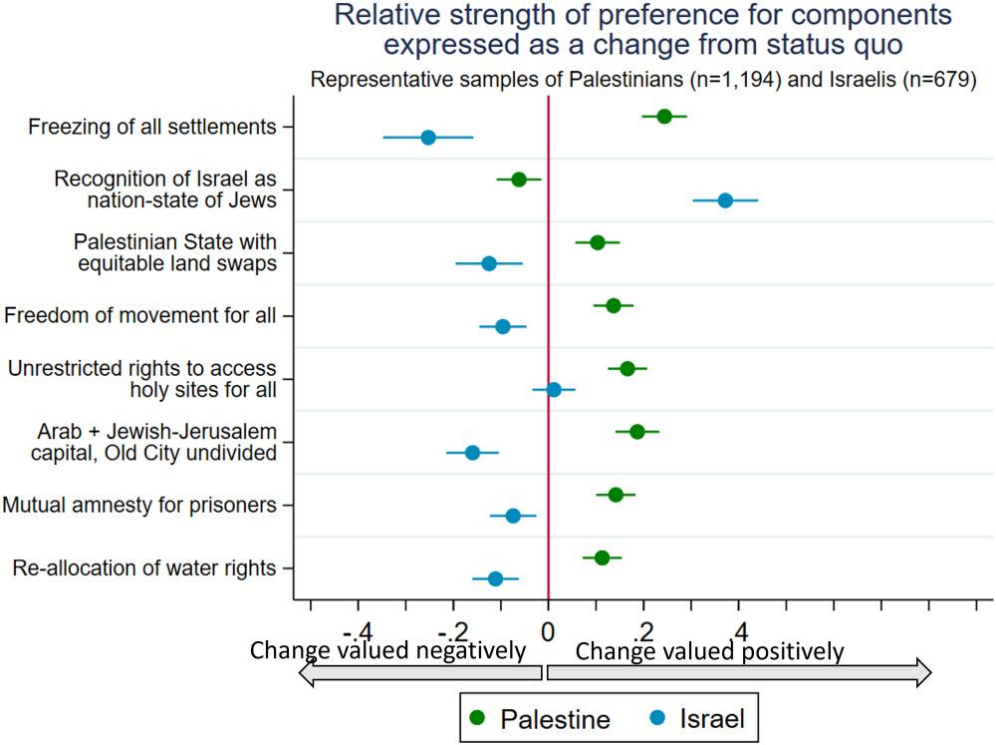
Strength of preferences for Israelis (blue) and Palestinians (green) for each of the eight components of prospective peace agreement expressed as the preference for a change from the status quo (zero).

Figure 2 displays the strength of preferences for Israelis (blue) and Palestinians (green) for each of the eight components of prospective peace agreement. These preferences are visualized as the preference for a *change* from the status quo, which is normalized at zero, and represents the alternative arrangements in column 1 of Table 1. The metric of the *x*-axis represents the desirability of each component: positive (negative) values indicates that the component being change from the status quo is valued positively (negatively), and thus increase (decrease) the acceptability of a deal. The horizontal lines indicate the 95% CI. For Israelis the most desirable component is “Palestinians recognizing Israel as the nation-state of the Jewish people.” For Palestinians the most desirable component is the “freezing of all settlement building.” Palestinians and Israelis value most changes from the status quo in opposing ways, as would be expected among parties in conflict. However, the results highlight at least one clear point of compromise: the component “unrestricted rights to access holy sites” is valued positively by Palestinians and is not viewed as detrimental by Israelis.

**Fig. 3.**
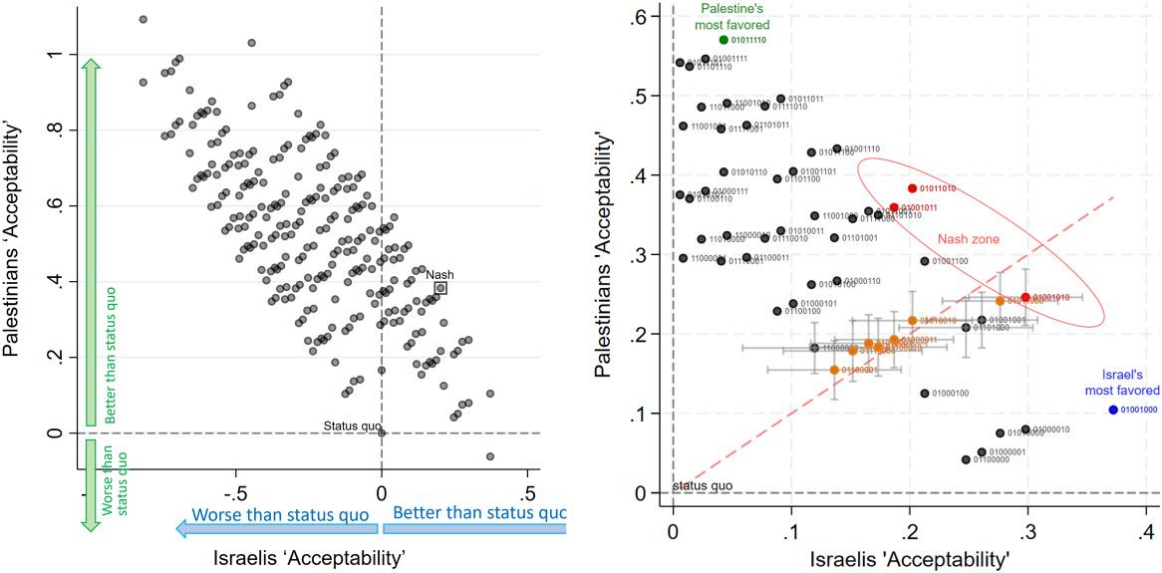
a) Acceptability of 256 prospective peace agreements for Israelis (*x*-axis) and Palestinians (*y*-axis). Point (0,0) is the status quo. b) Deals in the ZOPA. Labels indicates whether a component is changed from the status quo with “1” and a continuation of the status quo with “0.” The Nash zone groups the three deals with highest joint utility gains, $\Delta u_{Pj}^{0.5}\cdot \Delta u_{Ij}^{0.5}$ (red dots, within oval shaped curve). The deals in orange are “fair” deals that share utility gains equally.

Aggregating the strengths of preference for each component of the peace agreements yields a measure of the acceptability for each one of the 256 prospective peace agreements. Figure 3(a) maps the preferences for peace deals of Israeli and Palestinian people into the space for agreement. The point (0,0) indicates the status quo. The *x*-axis measures utility changes arising from each peace agreement compared with the status quo for Israelis. Positive values on the *x*-axis represents an improvement from the status quo and negative values represents a worsening. The *y*-axis measures the same for Palestinians. From the status quo, the North-East quadrant of the diagram (i.e. positive *x*- and *y*-axes) illustrates the set of peace deals that would be preferred over the status quo by both parties and, given the estimated preferences, are mutually acceptable to both sides. This is the ZOPA. The ZOPA between the two people is populated by 55 deals out of the 256 deal configurations that our design considers: these deals are preferable over the status quo for both parties. All other areas of the diagram contain deals that are unacceptable to at least one party.

Figure 3b provides a focused view of the ZOPA with each deal labelled as a sequence of “1”s and “0”s indicating that the relevant component is a change from the status quo (“1”) or a continuation of the status quo (“0”) ordered as in Table 1. Deals within the ZOPA that are furthest from the status quo increase acceptability for both parties. This means that deals positioned further northeast are preferred over the status quo; these deals are also preferred to other deals that are not as far away from the status quo.

Within the ZOPA, theoretical solutions suggest deals of interest as focal points embodying principles of efficiency and fairness. The Nash solution with equal bargaining power maximizes efficiency (i.e. maximizing the joint utility gain, $\Delta u_{Pj}^{0.5}\cdot \Delta u_{Ij}^{0.5}$) and represents a mutually desirable deal in the ZOPA that exhausts the “integrative potential” gains over the status quo. In our empirical application, we refer to deals closely approximating this solution as the “Nash zone.” In Fig. 3b, the three red-marked deals exemplify these options. As an illustration, the highest gains for both parties are achieved by a deal in the Nash Zone that has four components changed from the status quo: “Palestinians recognize Israel as a nation-state of the Jewish people,” “freedom of movement for people, vehicles and goods between the West Bank, Gaza and the State of Israel for both Palestinians and Israelis,” “unrestricted right to access the holy sites and freedom of worship for anyone,” “mutual amnesty and release for an agreed number of current prisoners” and the remaining components unchanged from the status quo: settlements building continues, the civil and military jurisdiction is like today, the Israeli capital in East and West Jerusalem and the Palestinian capital de facto in Ramallah, today’s unequal distribution of water rights. These components made up a deal configuration reminiscent of the confederal model as a framework for resolving the Israeli-Palestinian conflict (26).

Assuming the metric of acceptability is comparable between Israelis and Palestinians, deals that lie close to the 45 degree line of the ZOPA are all characterized by the property of fairness: these deals share gains from compromise evenly among the two parties.^k^ Deals furthest away from status quo have higher acceptability from each party’s perspective and thus can be considered preferable in a negotiation setting. We consider deals “close” if the 45 degree line is < 1 SE from the location of the deal in the ZOPA. Figure 3b shows them in orange. These deals have two or three components changed from the status quo. Among these deals, the deal displaying “An independent Palestinian state with equitable land swaps” and “per capita water rights” alongside “Palestinians recognizing Israel as a nation-state of the Jewish people” (and all other issues unchanged from the status quo, deal 01100001) is less preferred by *both* parties compared with an agreement in which “Palestinians recognize Israel as a nation-state of the Jewish people” and the “freedom of movement between Gaza, West Bank and Israel for everyone” and “unrestricted right to access holy sites for anyone” are guaranteed (deal 01011000).

All deals in the ZOPA include “Palestinians recognizing Israel as a nation-state of the Jewish people.” Deals that include “freezing of all settlement building” are favored by Palestinians and lie above the 45 degree line; while deals favored by Israel and below the 45 line have at most one concession to Palestinians.

## Does violence facilitate or hinder compromise?

In an ongoing conflict, understanding how direct or indirect experiences of violence influence the perspectives of individuals on prospective peace agreements is crucial. To capture these individual experiences, we crafted a bespoke questionnaire tailored to discern whether the respondent, any of their family members, friends, or acquaintances were victim of an incidence of violence related to the conflict, the timeline of the incident, and its outcomes (e.g. whether a person died, remained physically impaired, remained traumatized, or recovered). We were able to collect this information exclusively on the Israeli sample due to contractual constraints on the length of the survey on the Palestinian side. For Palestine, we use the geographical residence of the respondent, the Gaza Strip or West Bank, to distinguish different levels of exposure to violence related to the conflict. Gaza has four times the number of casualties compared with the West Bank in the period 2008–2022:^l^ this means that, once the population count is taken into account, there is roughly a six times higher probability of casualties in Gaza compared with the West Bank. However, the areas of West Bank and Gaza Strip differ in other socio-economic and political aspects, thus the results cannot be attributed to violence exposure alone.

**Fig. 4.**
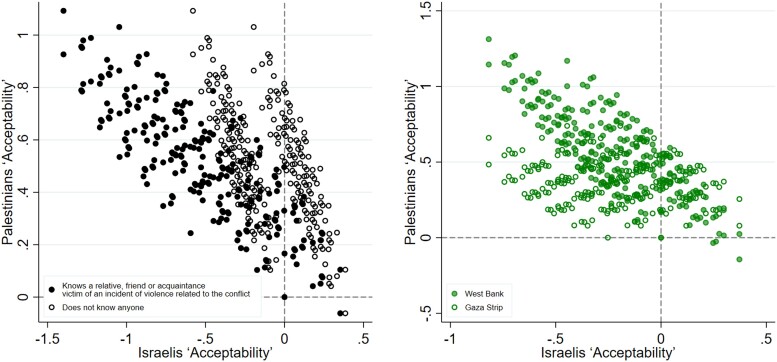
ZOPA by exposure to violence among a) Israelis and b) Palestinians.

Approximately 6.2% of the Israeli sample report being victim of an incident of violence related to the conflict with the Palestinians. A total of 30% report knowing someone who was a victim. Out of this 30% nearly half of the incidents (42%) concerned a person who died. Reported incidents occurred between 1986 and 2022 (up to the time of the data collection), with the highest number of violent events recorded in 2022 (11%), 2021 (9%) and cumulatively during the years of the second Intifada (20% between 2000 and 2005, see Fig. S4). The victimized group is, as expected, demographically different from the nonvictimized: it includes more men, a higher proportion of residents in the Jerusalem district (which border the West Bank) and the Judea and Samaria area (i.e. Israeli settlements) and younger respondents (Table S4). Figure 4a shows a reduced ZOPA for victimized Israelis (black dots): only 23 deals are acceptable for this group, compared with 99 for the nonvictimized (hollow dots). The analysis of preferences (Fig. S5a) reveals that the deviation into non-ZOPA quadrants is primarily influenced by two components: the freezing of settlements and the arrangement over the capital. Victimized individuals express a significantly stronger aversion to these changes from the status quo compared with their nonvictimized counterparts, six times and twice as much, respectively. These differences do not disappear when we control for additional demographic heterogeneity by gender, age and Jerusalem and Judea and Samaria districts (Table S5). Wald tests reported in Table S5 show the differences in valuations of peace deals’ components by exposure to violence remain jointly significant across specifications.

Within the group of victimized people, those who report knowing someone who died tend to have stronger aversion to peace deals than the average individual (Fig. S5b). These latter results should be interpreted with caution because standard errors are large due to the small sample size of the subgroup who knows a casualty (n=85). Yet, the result is replicated in a larger (n=392) yet nonrepresentative sample of Israeli citizens (Fig. S5c). With these limitations duly noted, the results suggests violence negatively influences the willingness to compromise, with most traumatic experiences reducing it most.

For Palestinians, Fig. 4b shows the ZOPA is almost equally populated for Gaza and West Bankers, with 56 and 53 deals, respectively, with in some cases different configurations of deals being preferred. This is explained by the analysis of preferences: Gazans value all changes from the status quo positively, including the “recognition of Israel as the nation-state of the Jewish people,” albeit with significantly smaller strengths of preferences compared with West Bankers for “freezing of all settlement building,” “freedom of movement for people, vehicles and goods between the West Bank, Gaza and the State of Israel for both Palestinians and Israelis” and “Palestinian capital in Jerusalem’s Arab-majority neighborhoods and Israeli capital in Jewish-majority neighborhoods.” These results chime with the finding from Palestinian polls, which find Gazans historically being more supporting of permanent peace settlements and more critical of Hamas than West Bankers ((30), Figure 13). However, there is heterogeneity within these regions, likely due to other unobserved factors, including individual support for Hamas. Looking at the rank position of the status quo as an indicator of general willingness to support a peace deal by district in the West Bank and Gaza Strip, Table S6 shows there is some geographic variation: on average, districts with larger cities rank the status quo lower, implying a larger number of deals is acceptable. These differences are not explained away by demographic controls.

Further heterogeneity analysis highlights differences in the strengths of preference for each component of the peace agreement by gender and by age, and thus the ZOPA is shaped differently when conditioning the results on these subgroups. Figure S7 shows that the ZOPA is more largely populated for women than for men. This is due to the Israeli women having strengths of preferences that yield more scope for compromise: women have higher valuation of “Recognition of Israel as a nation-state for the Jewish people” compared with Israelis men (P-value<0.05) and value concessions that are seen positively by Palestinians in a less detrimental way (i.e. less negative valuations). Palestinian women and men have similar strengths of preferences, with women mildly preferring “unrestricted access” compared with men (P-value<0.10). When looking at the strengths of preference for components by age groups (18–29; 30–50, and 50+), the results show that the ZOPA is smaller for younger respondents compared with older respondents (Fig. S8).

## Conclusions

This study develops a method to reveal the ZOPA between parties in conflict. Using representative samples of Israelis and Palestinians we show that a ZOPA existed: out of 256 potential deals considered, 55 are valued superior to the status quo by both groups. The most favored deals by both parties include changes from the status quo that hold tangible benefits for the daily lives of the people involved. Elements such as freedom of movement for everyone, unrestricted access to holy sites for all, prisoner releases, and recognition of Israel as a nation state for the Jewish people emerge as common ground. Deals that include these components are generally valued more favorably than deals advocating the constitution of an independent Palestinian state with territorial gains. The ZOPA that we identify is conditional on the component levels that were presented to the respondents. It may be possible to find a larger or smaller ZOPA if different levels for the components were used, for example if fractional components, such as freedom of movement for a proportion of people rather than all people, were used. Whether the ZOPA would increase or decrease in size at these different levels, compared with the ZOPA in this study, would require an understanding of how utilities change on each side in response to changes in the component levels. Undoubtedly, this would be a fruitful extension of our work.

The findings also reveal that exposure to violence hampers the prospects of achieving compromise among Israelis, reducing the ZOPA to 29 deals. For Palestinians, people from Gaza, where historically violence has been higher, appear to value positively all changes from the status quo, including the recognition of Israel as a nation state for the Jewish people.

The findings from case studies are often challenging to generalize to other conflicts, particularly intractable ones, due to the unique circumstances of each situation. However, when examining public support for peace agreements in other conflicts, certain common themes tend to emerge. In our study, tangible changes with economic value for both communities are the most prominent elements of ZOPA deals. Similarly, peace agreements that include economic provisions tend to be more valued in other contexts as well. For instance, work on peace mediation among Greek and Turkish Cypriots found that offering direct benefits, such as monetary compensation, increased support for an agreement in both communities (31). This emphasis on personal gains is also evident in Colombia, where research showed that individuals living in high-conflict areas were more supportive of negotiations (32), likely due to the greater tangible benefits they would experience from peace. By contrast, provisions related to territorial rearrangements and control are often more contentious, as shown in the study of Greek and Turkish Cypriots, as well as in ours.

The key message from this paper is that, at the time of the study, Palestinians and Israelis both expressed a genuine desire for an agreement. It is difficult to determine if or how preferences may have shifted in the aftermath of October 7th and how these changes influence the ZOPA between the two sides that one would observe today. Only further research could uncover these changes. However, while the current relative preferences may have changed, the relevance of the components revealed in this study is likely to still hold significance today. Mutual access to religious sites, mutual release of prisoners, mutual recognition of the State of Israel and a future State of Palestine, and freedom of movement are all components that are still likely to increase the acceptability of future agreements today. Additionally, the higher propensity to compromise observed in women is likely to persist, as this is a well-established finding in the negotiation literature. The analysis underscores one major constructive step toward resolving this conflict: the cessation of all hostilities.

## Supplementary Material

pgae581_Supplementary_Data

## Data Availability

All data and codes used in the analysis are deposited in the Harvard Dataverse https://doi.org/10.7910/DVN/B717EO for the purpose of reproducing the analysis.

## Notes

a A video-demo of the task in English language, using a 16 deals instead of 8, is available here: https://www.youtube.com/watch?v=uaiO8pO_f3k.

b A simple example might be that in a set of three deals, A, B, and C, the binary choice may reveal that A is preferred over B and C, but it would not indicate how B compares with C unless that specific pair is also randomly selected; instead, the ranking approach taken here reveals all comparisons within the same set.

c For example, in rating tasks, knowledgeable respondents may interpret the value of a scale more precisely than those less familiar with the subject: an expert might see a clear distinction between a 9 and 10 for quality, while a nonexpert might not. Additionally, ratings in the middle of the scale, such as a 5 on a 0–10 scale, often attract attention due to their association with indecision.

d Our design explicitly reveals the percentage of people who consider the no-agreement status quo a preferable scenario over all other alternatives.

e The typical conjoint analysis with options from the full factorial combination selected at random only guarantees orthogonality at the sample level.

f Strictly speaking, we cannot say whether what we are calling preferences are strictly preferences for the outcome or also take into account the perceived cost of implementation. The preamble of the study makes no reference to the costs of implementation and asks people about the outcome they would prefer to see in an unconstrained way. For this reason, we think that the study captures the demand side aspects of the question, not the supply side costs.

g The assumption of Independence of Irrelevant Alternatives is not restrictive in our ranking task, as respondents are permitted to change their ranking multiple times until confirmation, ensuring that the introduction of additional alternatives does not constrain the relative preferences between two options.

h Palestinian Center for Policy and Social Research.

i iPanel, www.ipanel.com.

j A demo from pilot testing in English language is available on https://www.youtube.com/watch?v=uaiO8pO_f3k.

k Identification of fair deals as those along the 45 degree lines relies on the assumption of inter-group comparability between utilities of Israelis and Palestinians. Assumptions on inter-personal comparability of utility are commonly made in the egalitarian solution by (27), as explained in (28, 29). For further discussion, see Supplementary Material, Section F.

l
https://www.ochaopt.org/data/casualties.
