# Supplementary material for: Revealing the zone of possible agreement between parties in conflict: An application to Israeli-Palestinian peace agreements
Source: PNAS Nexus. 2025 Jan 21;4(1):pgae581. doi: 10.1093/pnasnexus/pgae581 (PMC11748125; doi:10.1093/pnasnexus/pgae581)
Supplement: pgae581_Supplementary_Data [file pgae581_supplementary_data.pdf]

SUPPORTING INFORMATION

Article: “Revealing the Zone Of Possible Agreement between parties in conflict: an application to Israeli- Palestinian peace agreements”
by Elisa Cavatorta, Ben Groom and Gilead Sher

DRAFT

A. Reasons for choosing 8 binary components. The choice of 8 binary components is a trade-offs between the ability to estimate the desirability of each component separately and unconfounded, and feasibility tests with respondents on the field. Using a fractional design in 8 blocks of 8 deals, each with 8 binary components, allows to achieve Resolution IV in which no main effects are confounded with any other main effect or 2-factor interactions. Four main effects are potentially confounded with 3-factor interactions, the effect of which is commonly assumed null. These components are: right to access the holy sites, the location of capital cities, treatment of prisoners and allocation of water rights. Adding a nineth component would have compromised identification: some main effects which would have been confounded with 2-factor interactions. Opting for a design in which main effects are confounded with 3-factor interactions (Resolution IV) is typically preferable compared to selecting a design where main effects are confounded with 2-factor interactions (Resolution III).

Moving from binary components to 3 (or more) category components rapidly increases the total number of peace deals and thus it increases the sample requirements and the number of deals each respondent is required to rank, increasing cognitive burden and time of task completion. As an example, if we were to include 3 categories, instead of two, for only two components the total number of potential peace deals would more than double: 2^6 x $3^2 = 576$ instead of 256. If we had 8 blocks, each respondent would have been required to rank 18 deals. While the number of blocks could have, in principle, been increased to reduce the number of deals each respondent faced, using random blocks was already considered a significant complication by the enumerators on the field. Using a large number of blocks would have been impractical and posed the risk of jeopardising the quality of data with mistakes.

B. Power analysis. For the purpose of the power calculations, the ranking task can be seen as an 'exploded' choice experiment in which the ranking of the 8 peace deals consists of a number of decisions between different alternatives. (23) This allows us to calculate the power according to the approach outlined in (33, Section 4) for binary choice experiments. If a respondent has to rank n cards, there are $(n(n-1))/2$ pairwise comparisons possible and all of these would be required in order to reveal the complete ordering of the n cards. This means that 36 pairwise comparisons would be required to be equivalent to our ranking task of 9 deals. Table SI.1 shows the sample size calculation for an orthogonal design with 36 pairwise comparisons of peace deals. This might be an overestimate of the required number of paired comparisons if preference transitivity is assumed. Therefore Table SI.2 shows the power calculation for an orthogonal design where each person faces 18 pairwise choices. In the former case the sample size required to be able to detect an effect size of 0.05 (0.1, 0.15) at 5% significance level in at least 80% of the cases is 289 (73, 33). In the latter the sample sizes are 583 (148, 67). Our sample sizes are therefore sufficiently powered for these effect sizes.

α	$1-\beta$	ES = 0.05	ES = 0.1	ES = 0.15	ES = 0.2	ES = 0.3
0.10	0.8	211	54	24	14	7
0.10	0.7	152	39	18	10	5
0.10	0.6	110	28	13	7	4
0.05	0.8	289	73	33	19	9
0.05	0.7	220	56	25	15	7
0.05	0.6	168	43	19	11	6
0.01	0.8	469	119	54	32	15
0.01	0.7	380	96	44	26	12
0.01	0.6	311	79	36	21	10

Table SI.1. Minimum sample size to obtain power $1-\beta$ when testing at significance level $1-\alpha$ from an orthogonal design with 36 pairwise choices

α	$1-\beta$	ES = 0.05	ES = 0.1	ES = 0.15	ES = 0.2	ES = 0.3
0.10	0.8	425	108	49	28	14
0.10	0.7	307	78	35	21	10
0.10	0.6	222	56	26	15	7
0.05	0.8	583	148	67	39	19
0.05	0.7	444	112	51	30	14
0.05	0.6	340	86	39	23	11
0.01	0.8	946	240	109	63	31
0.01	0.7	766	194	88	51	25
0.01	0.6	627	159	72	42	20

Table SI.2. Minimum sample size to obtain power $1-\beta$ when testing at significance level $1-\alpha$ from an orthogonal design with 18 pairwise choices

DRAFT

C. Reasons for choosing the components' topics and levels. Since the focus was on citizens preferences, the components' reflect a selection of issues 'on the ground' that are considered important by Palestinians and Israelis themselves. For this reason, we prioritize issues perceived as important for the quality of citizens' daily life over issues related to the diplomatic process or international politics (e.g. the role of international mediators, external guarantees, membership of international organizations, ending of Israel boycott, etc.).

The selection of issues was guided by available data in the Peace Index and the priorities identified in the Palestinian-Israeli Pulse data: a joint poll conducted by the Palestinian Center for Policy and Survey Research and the Evens Program in Mediation and Conflict Management at Tel Aviv University. For example, in September 2018, the Peace Index found that 83% of Jewish-Israelis think "the Palestinians must recognize Israel as the nation-state of the Jewish people before peace talks with them can be revived".(34) The changes from the status quo on settlements, borders and access to holy sites was informed by past peace proposals and consultation with negotiators. The water distribution issues was informed by research in warfare ecology and consultation with Prof Michael Mason (35).

The choice of levels and related wording was also guided by experts' comment we received, the opinion of one of the authors, who has been an official peace negotiator, methodological reasons and clarity of the wording for respondents. For example, for the component related to the issue on Israeli settlements, we use a pragmatic resolution frequently considered in previous peace proposals: 'freezing the construction of new Israeli settlements, settlements adjacent to the 1967 line will become part of Israel and West Bank east of the wall/fence will be evacuated'. For some components we used limiting levels: e.g. freedom of movement for *all* people rather than a given proportion of people. This choice, in addition of helping with respondents' comprehension, provides an interesting upper bound of the like (or dislike) of that component.

Two notable issues were not included among the list of eight components: a resolution on the (over 6 millions) Palestinian refugees living abroad and the issue is Israeli security. The decision regarding the situation of Palestinian refugees was guided by the results of Palestinian surveys which shows that the Palestinian refugee issue is not among the top-priorities in the mind of the people. In a 2018 survey of conditions required by Palestinians to support a peace agreement with Israel, the condition that 'Israel acknowledges responsibility for refugee problem' ranked last out of 10 conditions (36, p.8). In 2020, studying the hierarchy of priorities of demands on each sides, the survey findings show again that only between 6-7% of Palestinians selected the request to 'allow Palestinian citizens, such as refugees, to live in Israel without becoming Israeli citizens' in exchange for various Israelis demands (37, p.21).

The exclusion of a component focusing on Israeli security was methodological. At the time of the survey, the Palestinian-Israeli security cooperation was in place and Israel controlled border crossings, airspace and sea waters. This security cooperation arrangements and Israel control represents the status quo. Looking at previous peace proposals, the most reasonable expectation in any peace agreement proposal is that Israel would continue to maintain its security apparatus and a security cooperation with any future Palestinian State. In our design, unless the attribute on security could be conjugated as a change different from the status quo, the valuation of security would not have been an identifiable parameter.

Our design also omits the monetary dimension, which removes one common source of incommensurability of strength of preferences and potential taboo (38).

DRAFT

Freezing of all settlement building, evacuation of those inside the West Bank. Settlements adjacent to the 1967 line become part of Israel.
Explanation: The expansion of Israeli settlements in the West Bank and East Jerusalem will cease. Settlement adjacent to the 1967 line will be part of Israel. West Bank settlements east of wall/fence will be evacuated.

Israel's settlement building continues.
Explanation: Building of Israeli settlements in the West Bank and East Jerusalem continues at the same rate as in recent years.

Palestinians recognise Israel as the nation-state of the Jewish People.
Explanation: Recognition by all Palestinians (those currently living in Israel and those elsewhere) that the territories of Israel are the Land of the Jewish civilization, alongside non-Jewish minorities, with equal rights and duties.

Palestinians do not recognise Israel as the nation-state of the Jewish People.
Explanation: No recognition by Palestinians of the Jewish customs, religion and traditions of the State of Israel.

An independent Palestinian State over the West Bank, Gaza and East Jerusalem with equitable (1:1 in value) land swaps with Israel and no Israeli military presence.
Explanation: An independent Palestinian State is established as a single territorial unit and connected territories within the 1967 borders. Land swaps based on 1:1 value, with value based on size and economic factors.

The civil and military jurisdiction over Israel, the West Bank and Gaza remains as today.
Explanation: The administrative jurisdiction remains as today: Areas A, B and C in the West Bank, current jurisdiction for the Gaza Strip and in Israel. No land swaps compensation.

Freedom of movement for people (no checkpoints/permits), vehicles and goods between West Bank, Gaza and State of Israel for both Palestinians and Israelis.
Explanation : Free movement means removal of work permit system, checkpoints and other movement restrictions to allow people to work, travel and trade between the current territories of the West Bank, Gaza, Jerusalem and State of Israel.

Current freedom of trade between West Bank, Gaza and State of Israel. Permit system for labour and vehicles.
Explanation : Goods are free to move between Israel and the West Bank, there are restrictions of trade of goods to and from Gaza; work permit regime for labour movement (as of today).

Unrestricted right to access to holy sites and freedom of worship for anyone.
Explanation : Anyone (Jewish, Muslim, Christian or other) from Israel and the Palestinian territories can access and pray in all holy sites (including Temple Mount/Haram al-Sharif and the Holy Sepulchre).

Current restricted rights to access to holy sites and pray.
Explanation : Access and right to pray as today: e.g. Haram al-Sharif/Temple Mount: Muslims can pray and non-Muslims allowed to visit but not pray.

Palestinian capital in Jerusalem's Arab - majority neighbourhoods and Israeli capital in Jewish - majority neighbourhoods. Old City is undivided.
Explanation : The Arab majority neighbourhoods in Jerusalem will be under Palestinian control and form the capital of Palestine; Jewish-majority neighbourhoods in Jerusalem will be under Israeli control and be the capital of Israel. The Old city administered by a council representing Christians, Muslims and Jews.

Israeli capital in West and East Jerusalem and Palestinian capital de-facto in Ramallah.
Explanation : Israeli capital designated as being West and East Jerusalem and Palestinian capital being de-facto located in Ramallah.

Mutual amnesty and release for an agreed number of current prisoners in Israeli and Palestinian jails.
Explanation : An agreed number of Palestinian prisoners held by Israel and Israelis prisoners held by Palestinians will receive amnesty and be released.

Current practices of imprisonment, pre-trial detention and occasional prisoner release continue.
Explanation : The use of imprisonment, administrative (pre-trial) detention and prisoner release and prisoner exchanges continues as seen in recent years.

Water rights in proportion to the population: 60% Israel, 40% Palestinian Authority.
Explanation: The water from the aquifers is allocated proportional to the current population in the Palestinian territories (approx. 5m people) and Israel (approx. 9m).

Oslo II water rights (the same as today): 71% Israel, 29% Palestinian Authority.
Explanation: The water from the aquifers is allocated according to the Oslo II Accord (1995, Article 40): 71% Israel, 29% Palestinian Authority.

Fig. SI.1. Components and related descriptors given to respondents.

DRAFT

D. Data and national representativeness. Table SI.3 shows the descriptive statistics of the Israeli and Palestinian samples alongside the benchmark Census statistics of reference.

	Sample of Israeli citizens (n=679)	Population Statistics from CBS	Sample of Palestinians (n=1,197)	Population Statistics from PCBS
**Population group (%)**				
Arab Israelis	19.0	19.0		
**District of residence (%)**				
Jerusalem	10.7	11.2		
Northern	19.7	16.2		
Haifa	15.0	12.2		
Central	23.1	25.1		
Tel Aviv	15.0	17.4		
Southern	13.4	13.9		
Judea and Samaria	3.0	3.6		
West Bank			65.83	61.91
Gaza Strip			34.17	38.09
**Sex (%)**				
Male	46.5	48.7	49.83	50.50
Female	53.5	51.3	50.17	49.50
**Age (%)**				
Age (mean years of age)	42.9	44.7	37.46	36.54
Between 18-29 yrs old	26.8	25.5	35.93	41.94
Between 30-39 yrs old	21.8	19.6	24.29	21.98
Between 40-49 yrs old	17.5	17.5	16.67	16.01
Between 50-59 yrs old	13.4	13.6	13.32	10.95
Between 60-69 yrs old	11.1	12.1	6.70	5.51
Equal and above 70 yrs old	9.4	11.6	3.10	3.61

Table SI.3. Sample statistics and target population statistics. The table shows the descriptive statistics for the Israeli citizens sample (column 1) and target population statistics from the Central Bureau of Statistics of Israel (2019 data, column 2), available on https://www.cbs.gov.il/en/publications/Pages/2020/Statistical-Abstract-of-Israel-2020-No-71.aspx. We use table 2.3a (sex and age), table 2.19 (district), and table 28 (education). The table also shows the descriptive statistics for the Palestinian sample (column 3) and target population statistics from the Palestinian Central Bureau of Statistics (column 4), available on https://www.pcbs.gov.ps/pcbs_2012/Publications.aspx. We use table 2 (sex and age), table 20, and 21 (education) from the Census Final Results - Detailed Report Palestine 2017 (the latest Census data available), and table 2 from Census Final Results - Detailed Report West Bank 2017 and Census Final Results - Detailed Report Gaza Strip 2017.

DRAFT

E. Task and application interface. The Palestinian sample was collected via in-person interviews conducted in Arabic by trained enumerators hired by a professional poll company. The fieldwork used a nationally representative sampling frame. The task was implemented using physical cards, like the one in Figure SI.2.

To collect the data on the Israeli sample we designed a bespoke interactive online application. Two versions of the application were made available: one in Hebrew for Jewish-Israeli respondents and one in Arabic for Arab(Palestinian)-Israeli respondents. The data collection used the database of respondents of an Israeli poll company. The task interface looks like the one in Figure SI.3. Respondents were given written instructions to complete the ranking exercise and instruction videos always available to them throughout the task.

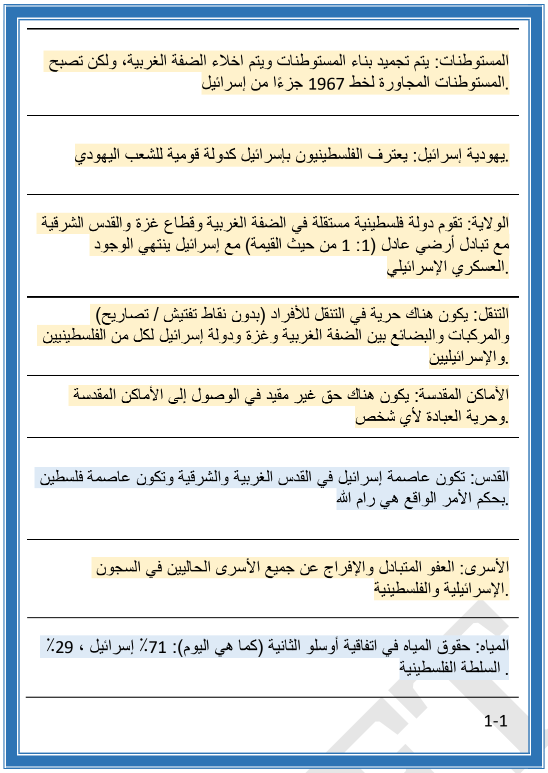

Fig. SI.2. An example of the cards representing peace agreements for the in-person fieldwork in Arabic language.

DRAFT

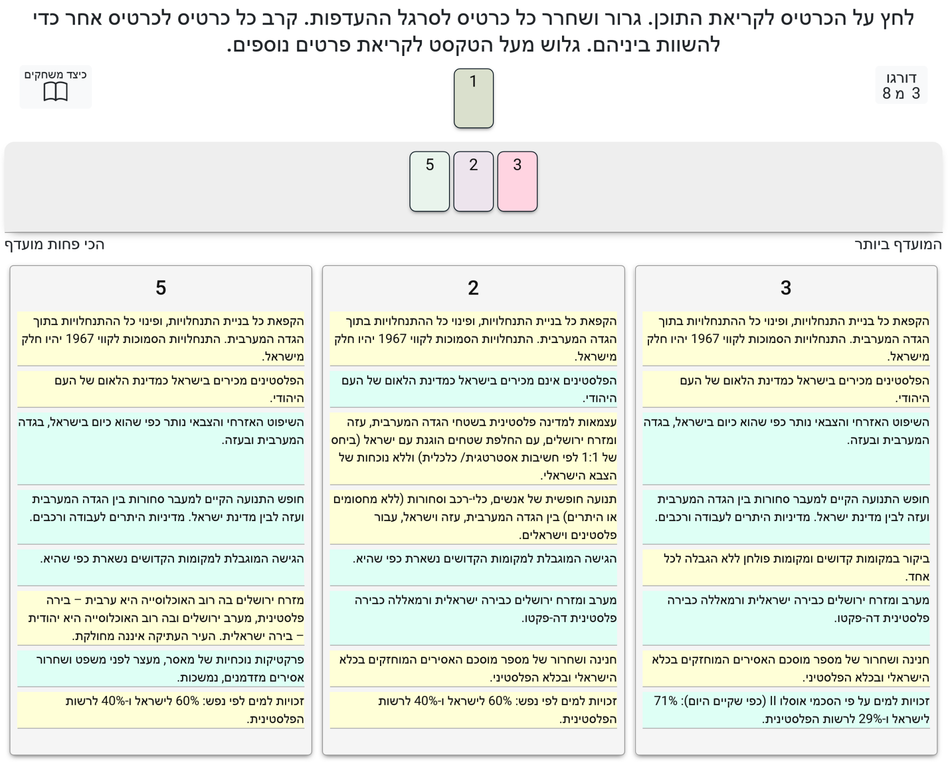

Fig. SI.3. An example of user interface of the online application in Hebrew language.

F. Considerations on the comparability of preferences. The model assumes that the individual rankings of peace agreements reflect ranking of preference/utility from peace agreements as in a Random Utility model. Utility of a deal j is assumed linear, $V_j(x) = x'_j\beta$, where x'_j is a vector of the agreement (binary) components – and separable in the contributions of each component. In the empirical model, the joint probability of a ranking is estimated as the product of logit probabilities: the estimated vector of parameters βs in the rank-ordered logit model can be interpreted as the expected *change* in utility for Israelis or Palestinians when a deal's component is changed from the status quo to an alternative arrangement.

Two linear utility functions are estimated, one for Israelis and one for Palestinians, and the two vectors of estimated βs are plotted in Figure 2 using a single metric: utility changes from the status quo. This process gives rise to two sets of considerations of commensurability/comparability of preferences: i) Between components; and, ii) Between Israelis and Palestinians.

Commensurability/comparability between components. Comparability between components means that if $\beta_m = 2\beta_k$, a change away from the status quo on component m is worth twice as much or is twice as desirable as a change from k. Under the assumptions made above, this statement is possible and components can be evaluated in the same metric. When preferences for components are aggregated into preferences for deals, commensurability between components implies that a deal that changes component m from the status quo compensates for the absence of a change from the status quo on component k if changes from the status quo in both m and k are valued positively.

The concern on commensurability/comparability between components arises when trade-offs between components cannot be done, for example because some component is considered a taboo (39) or an inviolable principle (40). It is worth noting that trade-offs among different dimensions of peace deals are an inevitable part of the process of negotiation. However, to shed light on the potential commensurability problem, we ask Israeli respondents to indicate whether conceding on the list of agreement's components (e.g. giving up the recognition of Israel as a Jewish state, conceiving the re-allocation of water rights between Israel and Palestinians under some mutually agreed criterion, etc.) was a list of 'inviolable principles, meaning that they can never be justified or be permissible under any circumstance, no matter what the material or human benefits, costs or consequences (e.g. no matter the monetary implications, efforts and resources required etc.)'. Only 2.5% of respondents indicated that the actions underpinning concessions on the eight components could never be justified.

Comparability between Israelis and Palestinians. It is known that utility functions are equivalent under positive affine transformation, that is $u'(x) = au(x) + b$, where a is a positive scale parameter and b is a translation/shift constant, and $u(x)$ reflect the same preferences. This implies that statements like 'Israelis prefer component X twice as much as Palestinians' are impossible to make because a and b for each group remain unidentified. As a result, mere differences in utility of a single deal between Palestinians and Israelis cannot be pinned down, because of scale, but differences in utility of a deal from a commonly valued deal, e.g. the status quo, can. We are merely concerned with these differences, hence the shift constant can be ignored. The differences in utility between each deal and the status quo are what is estimated by the rank-ordered logit model and then plotted in Figure 3. To identify the mutually acceptable deals and Pareto efficient deals in the sense of a Nash bargaining solution all that is required is the comparison of the utility of the peace deal with the utility of the status quo for each party. Ratios of differences in utility for Palestinians (P) and Israelis (I) can also be evaluated: $\frac{u^P(j)-u^P(sq)}{u^I(j)-u^I(sq)}$, for deal j and status quo sq, meaning that statements saying: 'it is X times as good to go from the status quo to deal j for Palestinians as to go from the status quo to deal j for Israelis' are reasonable.

Identification of fair deals as those along the 45 degree line in the sense that they achieve an egalitarian split of utility gains (27) rely on stronger inter-group comparability between Israelis and Palestinians, namely that the positive scale parameter a for each group is identical.

G. Heterogeneity by violence exposure, by gender and by age group.

DRAFT

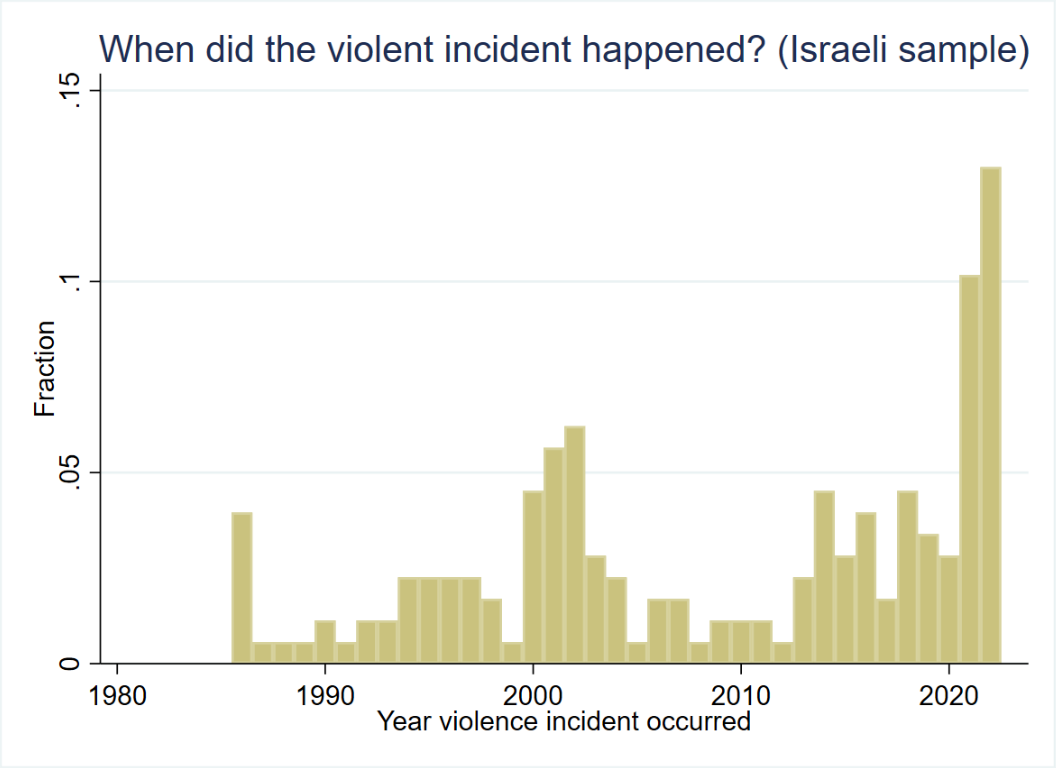

Fig. SI.4. Timeline of reported violent incidents, Israeli sample.

DRAFT

Demographic profile of Israelis exposed and not exposed to violence

	Know someone victim of an incidence of violence	Does not know anyone
Male (%)	52.76	43.96
Age (mean)	38.41	44.79
Aged $\leq$ 25 (%)	23.10	16.04
Arab (%)	16.58	20.00
Jerusalem (%)	18.09	7.71
Northern (%)	18.59	20.21
Haifa (%)	13.57	15.63
Central (%)	19.10	24.79
Tel Aviv (%)	13.07	15.83
Southern (%)	12.56	13.75
Judea and Samaria (%)	5.03	2.08

Table SI.4. Demographic characteristics of Israeli respondents who report knowing someone who was victim of an incident of violence related to the conflict with the Palestinians and those who did not know any victim.

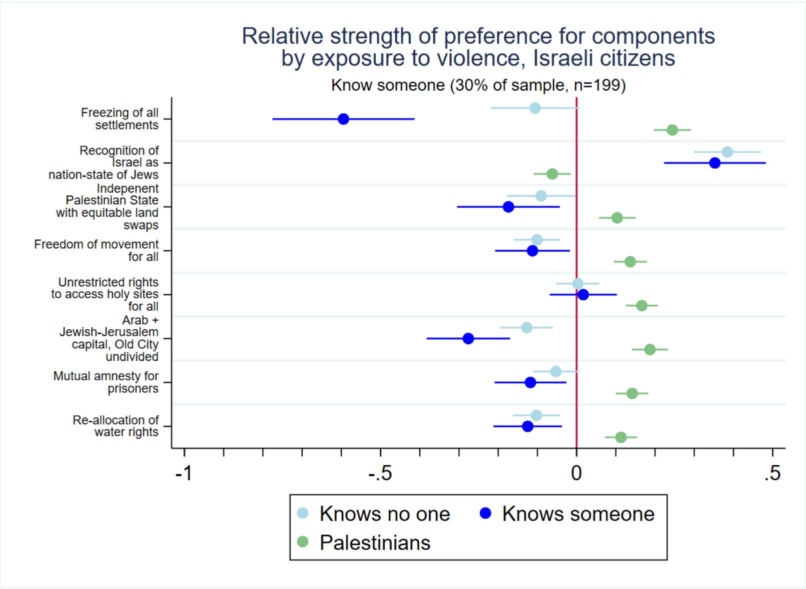

(a)

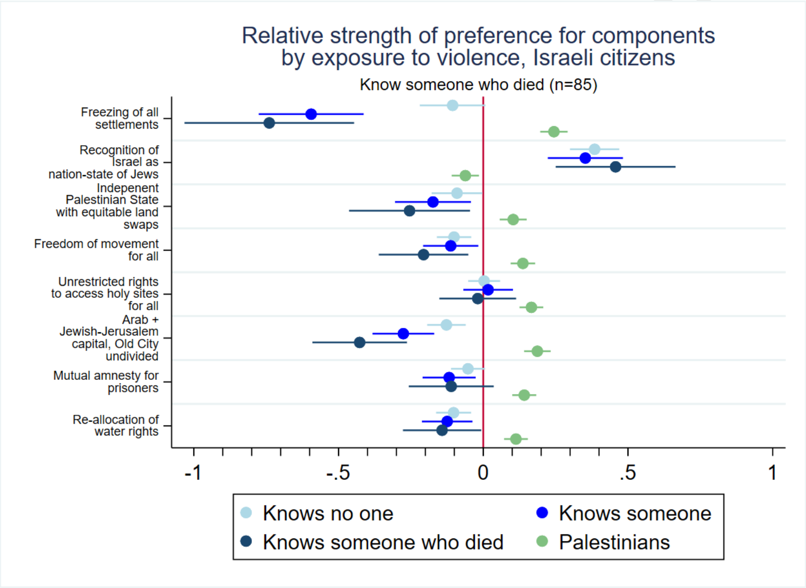

(b)

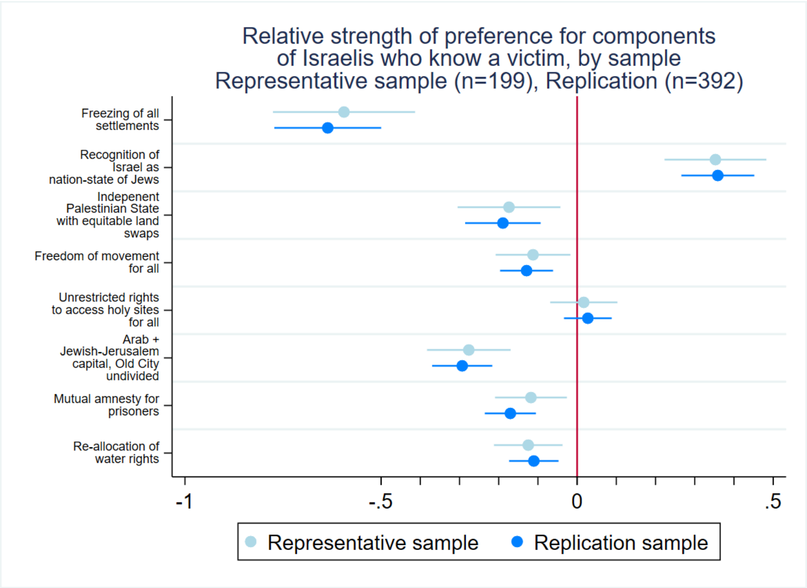

(c)

Fig. SI.5. Relative strengths of preference for components in sub-groups of (a) Israelis who know a victim or don't; (b) Israelis who know a victim, a victim who died or don't know anyone; (c) Replication of results in (b) using a non-representative sample of Israeli respondents.

Robustness of heterogeneity results by Exposure to Violence (EtV)

	Exposure to Violence	Additional controls (interacted with components)			
	heterogeneity	J+J&S district	J+J&S district<br>Age	J+J&S district<br>Age<br>Gender	Age<br>Gender
(a) Freezing of all	-0.1151**	-0.0645	-0.3162**	-0.1586	-0.2511*
settlements	(0.057)	(0.058)	(0.142)	(0.151)	(0.149)
(b) Recognition of Israel as	0.3847***	0.3915***	0.1568	0.2151*	0.2088*
nation state of Jews	(0.042)	(0.043)	(0.108)	(0.114)	(0.112)
(c) Palestinian state with	-0.1024**	-0.0917**	-0.2881**	-0.2469**	-0.2635**
equitable land swaps	(0.043)	(0.045)	(0.113)	(0.118)	(0.115)
(d) Freedom of movement for all	-0.0922***	-0.0952***	-0.2533***	-0.2295***	-0.2306***
	(0.030)	(0.031)	(0.078)	(0.082)	(0.080)
(e) Unrestricted rights to	0.0105	0.0128	-0.1223*	-0.0976	-0.0957
access holy sites for all	(0.027)	(0.028)	(0.073)	(0.076)	(0.074)
(f) Arab + Jewish Jerusalem	-0.1168***	-0.1074***	-0.2343***	-0.1927**	-0.2075**
Old City undivided	(0.033)	(0.035)	(0.086)	(0.089)	(0.086)
(g) Mutual amnesty for prisoners	-0.0575*	-0.0584*	-0.1598**	-0.1511*	-0.1478*
	(0.030)	(0.031)	(0.079)	(0.083)	(0.082)
(h) Re-allocation of water rights	-0.1140***	-0.1072***	-0.1252*	-0.1027	-0.1137
	(0.030)	(0.031)	(0.074)	(0.077)	(0.075)
EtV#(a)	-0.4794***	-0.4134***	-0.3827***	-0.3480***	-0.4046***
	(0.108)	(0.110)	(0.111)	(0.112)	(0.110)
EtV#(b)	-0.0319	-0.0255	0.001	0.0126	0.0097
	(0.078)	(0.081)	(0.082)	(0.082)	(0.081)
EtV#(c)	-0.0713	-0.0677	-0.0444	-0.0273	-0.0303
	(0.080)	(0.082)	(0.083)	(0.083)	(0.082)
EtV#(d)	-0.0202	-0.0118	0.0039	0.0066	0.001
	(0.057)	(0.059)	(0.059)	(0.060)	(0.059)
EtV#(e)	0.0064	0.0091	0.0284	0.0282	0.0279
	(0.052)	(0.052)	(0.053)	(0.054)	(0.053)
EtV#(f)	-0.1593**	-0.1461**	-0.1305**	-0.1127*	-0.1240*
	(0.064)	(0.064)	(0.065)	(0.066)	(0.066)
EtV#(g)	-0.0603	-0.0596	-0.0435	-0.0508	-0.0503
	(0.055)	(0.056)	(0.057)	(0.058)	(0.058)
EtV#(h)	-0.0107	-0.0007	0.0111	0.0083	-0.0006
	(0.054)	(0.055)	(0.055)	(0.056)	(0.055)
Assumption of constant valuation weights: Wald test's p-value of null of no heterogeneity by					
Exposure to Violence	0.0041	0.0246	0.0509	0.123	0.0446
Jerusalem district & Settlements		0.0919	0.1404	0.1369	
Years of age			0.0041	0.003	0.0018
Gender				0.004	0.0041

Table SI.5. Robustness of Exposure to Violence differences in preferences for deals' components controlling for heterogeneity in components by demographic characteristics listed at the top of each column: Jerusalem and Judea and Samaria district (J+J&S district), age (in years) and gender. Characteristics of the individual do not vary between alternatives and the average additive effect of these characteristics on the valuation of alternative deals cannot be identified, but interactions with components can. The panel at the bottom of the table reports the p-values of Wald test statistics of the null hypothesis that valuations of components do not vary (that is, there is no heterogeneity) by each characteristic (row) across the various specification (column).

DRAFT

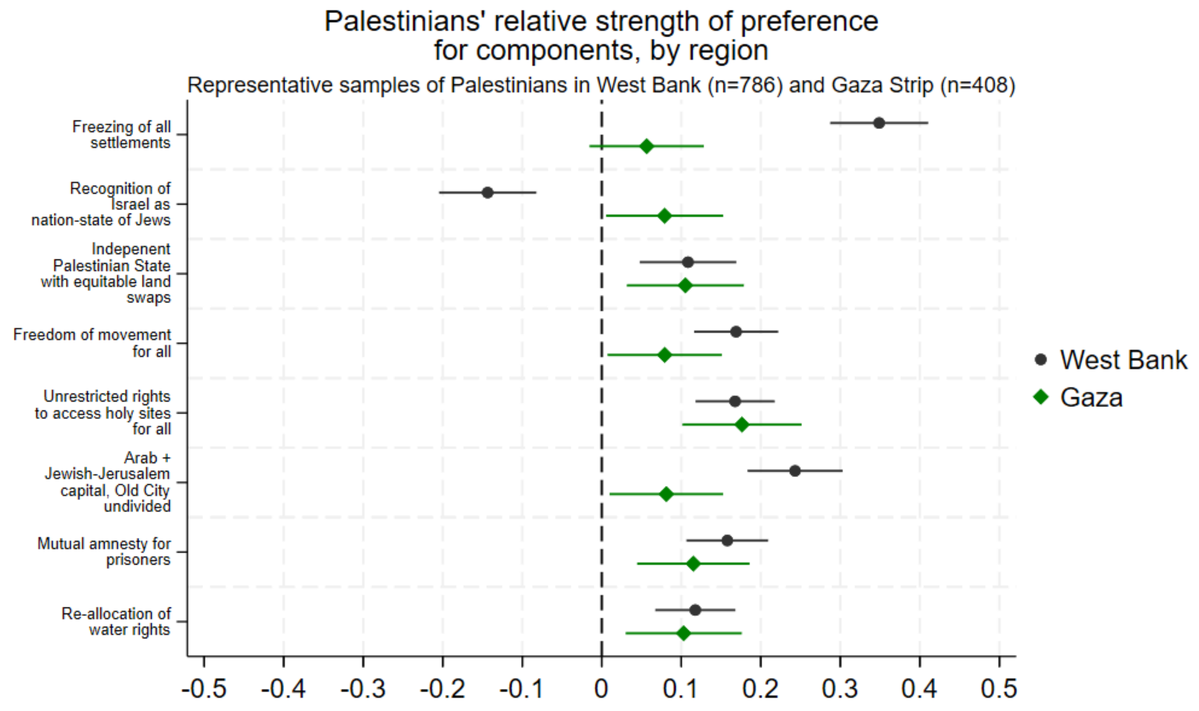

Fig. SI.6. Relative strengths of preference for components in sub-group of Palestinians by region.

DRAFT

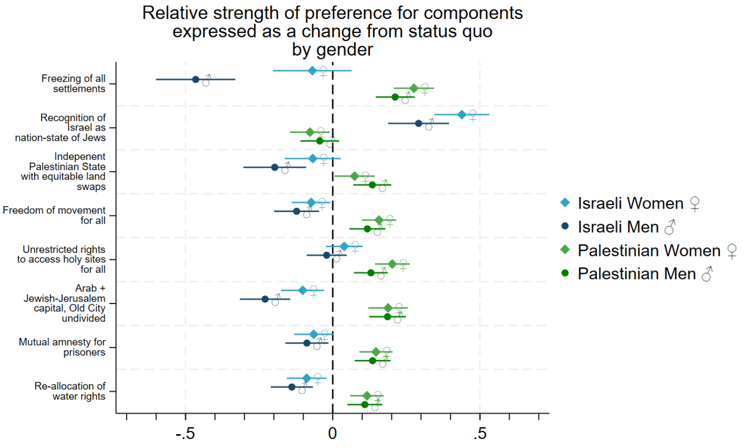

(a)

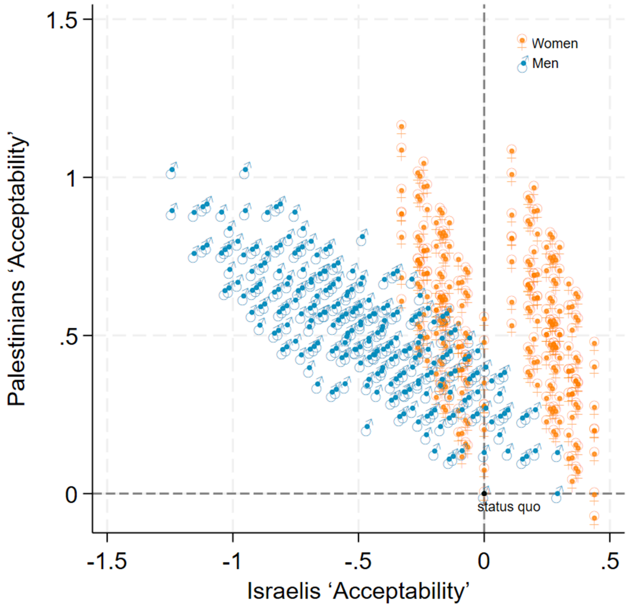

(b)

Fig. SI.7. (a) Relative strengths of preference for components in sub-groups of women and men on both groups; (b) Acceptability of 256 prospective peace agreements by gender.

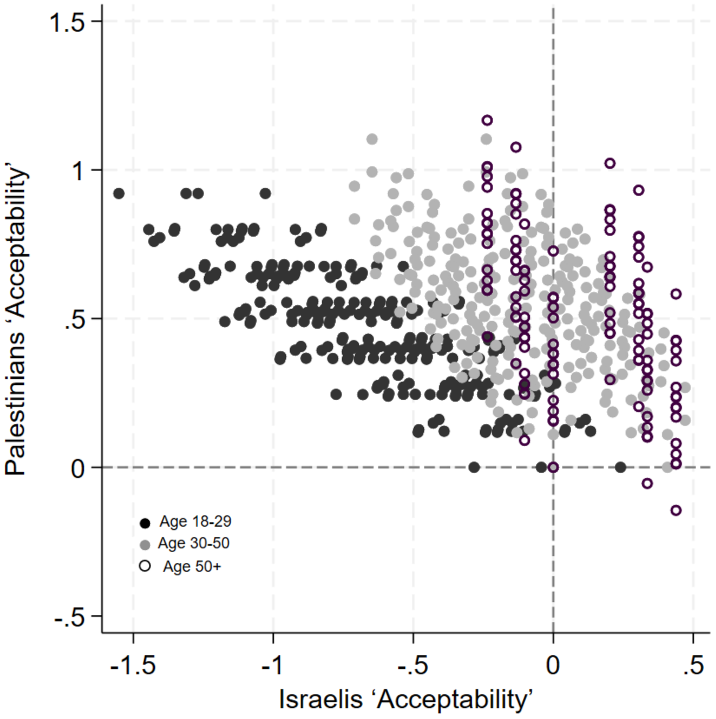

Fig. SI.8. Acceptability of 256 prospective peace agreements for Israelis (x-axis) and Palestinians (y-axis) by age group.

DRAFT

Outcome variable: Status Quo's rank position, Palestinian sample		
	Averages (s.e.) (1)	OLS Regression with controls (2)
Jenin	6.8101***	-0.9265**
	(0.2611)	(0.378)
Tubas	5.4250***	-2.3072***
	(0.483)	(0.462)
Tulkarem	5.6000***	-2.1856***
	(0.394)	(0.438)
Qalqilya	7.8500***	0.1022
	(0.483)	(0.428)
Salfit	5.0000***	-2.7340***
	(0.483)	(0.514)
Nablus	6.5111***	-1.2251***
	(0.322)	(0.378)
Ramallah	7.5114***	-0.2657
	(0.325)	(0.368)
Jerusalem	7.3578***	-0.4408
	(0.292)	(0.356)
Jericho	5.9250***	-1.7091***
	(0.483)	(0.505)
Bethlehem	6.8800***	-0.9021**
	(0.432)	(0.454)
Hebron	6.6733***	-1.0653***
	(0.249)	(0.353)
Jabalia	7.3676***	-0.3607
	(0.370)	(0.347)
Gaza city	7.7154***	reference
	(0.268)	
Khanyounis	5.5875***	-2.1458***
	(0.341)	(0.344)
Deir al Balah	5.2714***	-2.4451***
	(0.365)	(0.456)
Rafah	4.0667***	-3.7010***
	(0.394)	(0.402)
woman		-0.0682
		(0.146)
age		0.0049
		(0.005)
education level		0.0290
		(0.053)
subjective income class		0.0217
		(0.075)
constant		7.5446***
		(0.462)
R2		0.144
obs		1190

Table SI.6. Averages and OLS regression of Status Quo's rank position (i.e. 1 if most preferred, 2 if second-preferred, etc) on district indicators. Column 2 uses Gaza City as reference category. Coefficients are differentials compared to Gaza City. Robust standard errors are in parentheses. 'woman' is an indicator variable if the respondent is a woman. 'age' is years of age. 'education level' is an index of educational achievement. 'subjective income class' is an ordinal variable reporting how the respondent would rate their current household income in relation to the poverty line.

DRAFT
